# A Case Report of Fetus Papyraceus in Singleton Pregnancy

**DOI:** 10.3390/reports8040203

**Published:** 2025-10-13

**Authors:** George Stoyanov, Ivaylo Balabanov, Svetoslava Zhivkova, Hristo Popov

**Affiliations:** 1 Department of Pathology, Multiprofile Hospital for Active Treatment, 9700 Shumen, Bulgaria; 2 Department of Obstetrics and Gynecology, Multiprofile Hospital for Active Treatment, 9700 Shumen, Bulgaria; 3 Department of General and Clinical Pathology, Forensic Medicine and Deontology, Faculty of Medicine, Medical University—Varna, 9000 Varna, Bulgaria

**Keywords:** fetus papyraceus, singleton pregnancy, fetus compresus, miscarriage complication

## Abstract

**Background and Clinical Significance**: Fetus papyraceus is a term describing fetal findings associated with miscarriage, wherein the fetus is not expelled, remains in the uterine cavity, and is compressed by neighboring structures, with an inability for fetal resorption due to advanced pregnancy. **Case Presentation**: Herein, we present the case of a 33-year-old primigravida with two previous presentations to our institution due to emotional stress without evidence of physical abuse, the last one being at the 14th week of pregnancy. The latest presentation was with complaints of intermittent lower abdominal pain and an outpatient gynecology consultation describing fetal demise, with fetal parameters corresponding to demise in the 15th to 16th week of gestation. Pregnancy termination was performed with the specimen sent for pathology, revealing fragmented placental parts, which, on section, were firm, with greyish areas and notable calcification, fragments of an umbilical cord appeared normal, and a significantly compressed fetus, which was flattened in the anteroposterior aspect with significant compressive deformation of the limbs—fetus papyraceus characteristics. Histology of the placental fragments revealed fibrin thrombi in large blood vessels, intense fibrosis of the villi with focal fibrin extravasation, and focal necrosis and inflammation, as well as extensive calcium deposits. **Conclusions**: Fetus papyraceus is a rare complication of intrauterine demise and fetal compression, which can vary in its degree of presenting severity and requires the co-occurrence of specific conditions. The condition is rarely associated with singleton pregnancies.

## 1. Introduction and Clinical Significance

Fetus papyraceus is a term describing fetal findings associated with miscarriage, wherein the fetus is not expelled, remains in the uterine cavity, and is compressed by neighboring structures, with an inability for fetal resorption due to advanced pregnancy [1]. This intrauterine demise and compression, together with dehydration of the dead fetus, slowly lead to its flattening where the fetus gains a leather-like or parchment paper appearance and consistency [1,2,3,4]. In advanced cases, fetal calcification can also be noted. Synonym terms include fetal mummification, fetus compressus, and vanishing twin [2,3,4,5,6].

Fetus papyraceus is a rare condition and is almost always exclusively associated with twin pregnancy and has a reported incidence of 1 in every 17,000–20,000 pregnancies [1]. As twin pregnancies are the main condition associated with the condition, incidence in them is significantly higher and reported as 1 in around 200 twin pregnancies; however, their incidence is significantly lower than that of singleton pregnancies, as they represent around 3% of all pregnancies [1,3].

Fetus papyraceus is an exceptionally rare condition in singleton pregnancies.

## 2. Case Presentation

Herein, we present the case of a 33-year-old primigravida with a previous medical history of splenectomy as a child due to trauma. The patient initially presented to our institution at the 10th gestation week with complaints of intermittent abdominal pain after severe emotional distress. Blood pressure was 125/80 mmHg, no edema or elevated liver proteins were noted; the patient denied any physical trauma, and a fetal ultrasound revealed normal fetal parameters and a gestational age of 10 weeks. The patient was prescribed spasmolytics and referred for an outpatient follow-up.

The second presentation was four weeks later due to elevated blood pressure. On physical evaluation, blood pressure was 160/100 mmHg, and no edema was noted. Laboratory results revealed no elevated liver enzymes. The patient denied emotional distress and physical trauma. Fetal ultrasound revealed normal fetal parameters and adequate development compared to the previous presentation, with a gestational age of 14 weeks. The patient’s blood pressure normalized during the physical examination, and 30 min after presentation, it was 120/80 mmHg. The patient was advised to have regular outpatient visits with a gynecologist and to consult a cardiologist for ongoing blood pressure monitoring.

The third presentation to our institution was one month later (18–19th gestational week). The patient complained of intermittent lower abdominal pain, and an outpatient gynecology consultation revealed fetal demise during ultrasound, with fetal parameters corresponding to demise in the 15th to 16th week of gestation. Laboratory tests were within normal parameters, apart from a slightly decreased hemoglobin—116 g/L (laboratory reference 120–160 g/L); red blood cell number—4.03 × 10^12^/L (laboratory reference 4.2–5.4 × 10^12^/L); and hematocrit—0.34 L/L (laboratory reference 0.36–0.48 L/L). Inflammatory markers and coagulation were completely within normal range; only monocytes were slightly increased—1.71 × 10^9^/L (laboratory reference 0–1 × 10^9^/L). Urinalysis was within reference parameters.

Due to the fetal demise, emergency uterine evacuation was performed under general anesthesia by means of cervical dilation and manual extraction, with complete removal of the fetus, umbilical cord, and placenta per vaginam, and the specimens were sent to pathology for evaluation. The post-evacuation period was uneventful, and the patient was discharged on the third post-evacuation day.

**Figure 1:**
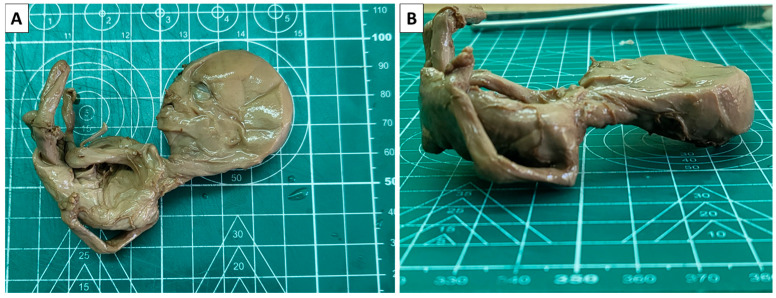
Fetus papyraceus. (**A**): superior view; (**B**): lateral view.

Gross pathology of the specimens revealed fragmented placental parts, which, on section, were firm, with greyish areas and notable calcification. Fragments of an umbilical cord appeared normal, and a significantly compressed fetus, which was flattened in the anteroposterior aspect, showed significant compressive deformation of the limbs (Figure 1).

**Figure 2:**
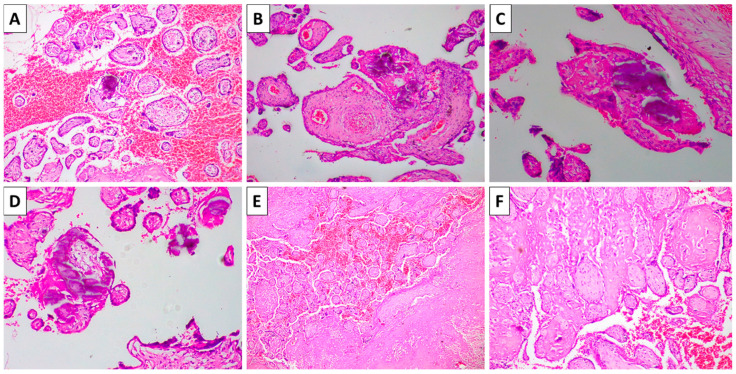
Histology of the placenta. (**A**): Hemorrhage and villous calcification, H&E stain, and original magnification 40×; (**B**): fibrosis of the placenta and villi with focal calcifications, H&E stain, and original magnification 100×; (**C**): villous fibrosis and calcifications, H&E stain, and original magnification 200×; (**D**): villous fibrosis and calcifications, H&E stain, and original magnification 100×; (**E**): fibrous change in the placental stroma with focal necrosis, fibrin extravasation and hemorrhage, H&E stain, and original magnification 40×; and (**F**): fibrous change in the placental stroma with focal necrosis, fibrin extravasation and hemorrhage, H&E stain, and original magnification 100×.

Histology of the placental fragments revealed fibrin thrombi in large blood vessels, intense fibrosis of the villi with focal fibrin extravasation, and focal necrosis and inflammation, as well as extensive calcium deposits (Figure 2). None of the changes corresponded to typical changes for the gestational week. Thrombosis, necrosis, and inflammation were attributed to intrauterine retention, while fibrosis, fibrin, and calcium deposits were attributed to exaggerated placental aging. Fetal changes were attributed to intrauterine demise, retention, and compression for a prolonged period, leading to the fetus papyraceus transformation.

## 3. Discussion

Fetus papyraceus requires a unique combination of conditions for its development. Mainly, the gestation age of fetal demise must be between the 10th and 20th week of pregnancy, which typically does not allow for fetal expulsion or resorption, and the fetus is small enough to not cause any significant clinical symptoms [1,2]. This, in turn, allows for its slow compression, typically by its surviving twin, dehydration, and following mummification [1,2]. In a set of cases, the following degenerative cases can be so severe that the fetus undergoes calcification or even a membranous transformation to the point that it can be identified only in histological evaluation of the placenta and membranes [1]. In such instances, the mummified fetus is histologically represented by a focal thickening of the placenta, with extensive calcification and even ossification of the remnant fetal tissues [1]. In these instances, especially in a late presentation of the pregnancy or in unfollowed pregnancy, the presence of the mummified twin is often missed antepartum [1,2].

As seen in the presented case, while initially developing normally for its gestational age up until the 14th gestational week, by the time fetal demise was noticed (18–19th gestational week), ultrasound determined that the fetal age corresponded to the 15–16th gestational week. This indicated intrauterine retention for 2 to 4 gestational weeks in utero.

The condition has long been known to humanity, with some sources stating that depictions date back as far as Pliny the Elder in the first century AD [7]. More modern depictions were made by Ramsbottom in the 19th century, who states that the condition was mentioned in medical texts as far back as the 16th century [1]. The term fetus papyraceus itself was coined in 1872 by Settegast, with the French trivially calling the condition un petit bonhomme du pain d’épice—the little gingerbread man [1,7].

Unlike in most reported cases and the classical depiction of fetus papyraceus, in our case, it is not a twin, but a singleton pregnancy. Hence, the mechanisms for development, despite following the same pathophysiological steps, have a different etiopathogenesis. The main difference in etiology is the lack of a twin slowly compressing the deceased fetus [4,8]. In our case, first of all, the miscarriage went unnoticed by the mother; second, it produced either no or only mild and insignificant symptoms and was accompanied by uterine dystonia and a lack of cervical dilation, not allowing for proper contractions and attempts of fetal expulsion. Uterine contraction was probably gradual and mild, allowing for the compression of the limbs and overall fetal flattening of the deceased fetus until the misarrangement was noted on the outpatient’s ultrasound.

In our literature search, we were able to identify only one other instance of a reported fetus papyraceus in a singleton pregnancy, presented at the 15th World Congress in Fetal Medicine in 2016 [4]. Unlike in our case, wherein the mother was a primagravida, in the report by Inan et al., the mother is a multigravida (gravida 5, para 3, abortion 1) [4]. In the reported case, once again, however, the pregnancy was singleton and, as per last menstruation, was in the 33rd week of gestation; however, the ultrasound measurements showed a fetal age of 12 weeks, with a previous ultrasound performed at the 9th week of gestation and showing a vital fetus corresponding to the term of pregnancy [4]. Other reports only sparsely state that, while prevalent in twin pregnancies, the condition can also be observed in singleton pregnancies as well [1,2,3].

Other potential mechanisms not present in our case, which may lead to similar retention of a demised fetus, for which we were unable to identify references of feral mummification include hymen imperforata, previous uterine surgical intervention (such as previous caesarian section), or myoma.

Currently, other than twin pregnancies or pregnancies with more than two twins, there are no known risk factors for the development of fetus papyraceus [9,10]. There are, however, multiple reports of fetus papyraceus being associated with aplasia cutis congenita [10,11,12,13,14]. Aplasia cutis congenita is in itself an idiopathic condition in which there is a congenital absence of skin in certain areas of the body [10,11,12,13,14]. The two conditions often coexist and are thought to be closely linked with one another, mainly through infection, teratogens, fetal transfusion, or medications [10]. Of note, fetal transfusion in certain cases can be the leading cause of fetal demise of one of the twins [14]. Conversely, aplasia cutis congenita in the form observed in correlation with fetus papyraceus may be an incidental finding developing from the in utero presence of the demised twin, as it flattens due to the growth of the surviving twin; this may also have an effect on the survivor in the compression of certain areas of the body and the development of decubitus-like lesions in the areas where the twins are touching [14].

## 4. Conclusions

Fetus papyraceus is a rare complication of intrauterine demise and fetal compression, which can vary in its degree of presenting severity and requires the co-occurrence of specific conditions. The condition, despite being known for centuries, is a rare one and almost always associated with twin pregnancy. In the currently depicted case, an evident fetus papyraceus change was noted in a singleton pregnancy.

## Data Availability

The original contributions presented in this study are included in the article. Further inquiries can be directed to the corresponding author.

## References

[1] Dharwadkar A., Viswanathan V., Vimal S., Iqbal B. (2023). Fetus Papyraceus in Twin Pregnancy: A Rare Case Report. J. Sci. Soc..

[2] Lau W.C., Rogers M.S. (1999). Fetus Papyraceous: An Unusual Cause of Obstructed Labour. Eur. J. Obstet. Gynecol. Reprod. Biol..

[3] Dahiya P., Bains R. (2014). Conservative Management of Fetus Papyraceus: A Report of Two Cases. Oman Med. J..

[4] Inan C., Sayin N., Erzincan S., Uzun I., Sutcu H., Varol F. A Case of Fetus Papyraceus. Proceedings of the 15th World Congress in Fetal Medicine.

[5] Chikhale M.V., Pradhan P. (2019). Incidentally Diagnosed Fetus Compressus on Placental Examination. J. Postgrad. Med..

[6] Luna L., Barrahan R., Cruz H. (2011). Fetus Compressus and Fetus Papyraceous. Clinical Differences (Report of Three Cases). Ginecol. Obstet. Mex..

[7] Masamatti S.S., Ramteerthakar N.A., Pandav A.B., Gosavi A.V. (2015). Fetus Papyraceus: A Rare Case Report. Ann. Pathol. Lab. Med..

[8] Neto A., Ferreira A.P., Guimarães A.E., Vieira N. (2024). Aborto unifetal em gestação gemelar com presença de feto papiráceo. Rev. Científica Cerem-Go.

[9] Gulati G., Das B., Deepika D. (2015). Twin Foetus Papyraceous in Triplet Pregnancy. Int. J. Reprod. Contracept. Obs. Gynecol..

[10] Simic D., Prohić A., Puizina-Ivić N., Zeljko-Penavić J., Tomić T. (2015). Aplasia Cutis Congenita in a Newborn Child Associated with Two Fetus Papyraceous. Acta Dermatovenerol. Croat..

[11] Ws S., Tgm P., As A., Aahs A., Rms P. (2021). Case History Neonate with Aplasia Cutis Congenita Associated with Foetus Papyraceus. Sri Lanka J. Perinat. Med..

[12] Souarji A., Saoud S., Oufkir A.A. (2024). Aplasia Cutis Congenita Associated with a Fetus Papyraceus: A Case Report. Indian J. Paediatr. Dermatol..

[13] Snyder M.L., Ilyas H. (2019). Type V Aplasia Cutis Congenita with Fetus Papyraceus. JAAD Case Rep..

[14] Uzuner C., Seeho S.K.M., Smith C.J. (2017). Aplasia Cutis Congenita with Foetus Papyraceus: Case Report and Review of the Literature. J. Obstet. Gynaecol. Lahore.

